# Sediment Remediation for Ecosystem in Eutrophic Lakes

**DOI:** 10.1100/tsw.2002.156

**Published:** 2002-04-05

**Authors:** Y. Amano, K. Taki, K. Murakami, T. Ishii, H. Matsushima

**Affiliations:** Chiba Institute of Technology, 2-17-1 Tudanuma, Narashino-shi, Chiba 275-8588, Japan; Nihon University, Kanda Surugadai, Chiyoda-ku, Tokyo 101-8301, Japan

**Keywords:** remediation, eutrophic sediment, dissolved air flotation, chemical remediation materials

## Abstract

The remediation method — namely, a hybrid system combined with DAF and CRM — is studied in this paper for the size reduction of aqua-ecological circulation and for the elution control in lakes. Results show that two effects on water quality purification, the sediment washout effect and the elution control effect, can be induced by this system, and the biota inhabiting the lake is therefore shifted into an oligotrophic aspect, from blue algae to green algae and/or diatoms.

### INTRODUCTION

Expanded and distorted ecological circulation appears in eutrophic lakes, which are inhabited by phytoplankton such as water bloom, in conditions of irregular growth[1]. Eco-circulation is generally increased by nutrient salts, which are washed into a lake by river flow, including domestic wastewater, and eluted from the sediment layer as a detritus of the planktons and organic matter. Furthermore, the cause of distorted autochthonous eco-circulation is an over accumulation of nutrient salts, and a distortion of species/quantity of the aquatic biology is observed in a lake as a result[2].

A hybrid method derived from Dissolved Air Flotation (DAF) and Chemical Remediation Materials (CRM) is applied for the remediation in this paper. Individually, DAF and CRM are useful methods for reducing the amount of ecological circulation and for elution control. The hybrid method in this paper is conducted in low efficiency because the renovation of the ecosystem must proceed slowly into the normal/quasi-oligotrophic condition in regard to the nutrient salts. Minimizing the damage for varying quantities depends on changing the speed of the aquatic biology species/quantity[3].

### EXPERIMENTAL PROCEDURE

The experiment is conducted within a narrow range from 15 to 17% of the ignition loss which is in natural sediment condition of urban lakes. The sediment in this study is classified in three treatment groups of eutrophicated matter: raw/nontreated sediment group, remedied sediment group with DAF method, and elution control group with CRM method.

#### Procedure on DAF Method

The apparatus of the DAF method shown in Fig. 1 is composed with three parts: a reactor with a volume of 50 l (height: 100 cm, diameter: 30 cm), a microbubble generator controlled at a pressure of 7 kgf/cm$^2$, and a coagulation tank. From the bottom of reactor, the polyferric sulfate coagulant (4 l of 200 mg l$^{-1}$) and the microbubbles are continuously injected into the reactor, which is filled by the wetted sediment of 7 kg and the lake water. The bubble diameter is about 3 μm.

Organic matters are separated as froth, and settling coarse particles are separated as sediment particles from the raw sediment by the manipulation of DAF apparatus, and the mitigated sediment is obtained from the bottom of reactor tank as coarse sediment.

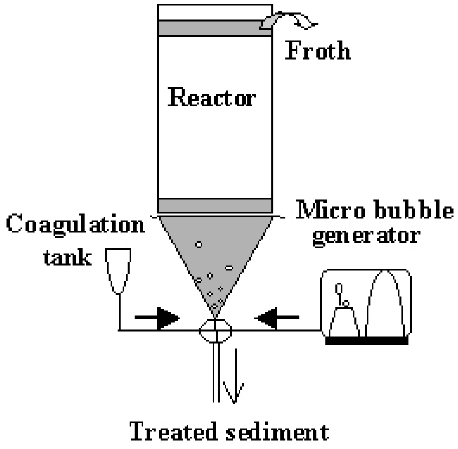

**FIGURE 1**. DAF Apparatus.

#### Elution Control with CRM Method

The CRM method with MgO controls the elution of nutrient salts from the sediment layer by combining MgO with phosphate ion to produce magnesium hydrogen carbonate, magnesium ammonium phosphate (MAP), or magnesium phosphate as shown in following equations.

$$4CO_2 + 3/2O_2 + 2MgO + 2NH_3 \rightarrow 2Mg(HCO_3)_2 + H_2O + N_2\uparrow \quad (1)$$

$$Mg^{2+} + NH_4^+ + HPO_4^{2-} + OH^- + 6H_2O \rightarrow MgNH_4PO_4{\cdot}6H_2O\downarrow + H_2\uparrow \quad (2)$$

$$H_3PO_4 + MgO + 2H_2O \rightarrow MgHPO_4{\cdot}3H_2O\downarrow \quad (3)$$

Here, the reaction in Eq. (1) is preferentially favored when the lake water is under redaction state, i.e., pH is in acid condition. In this reaction, it is very important for the CRM method that the elution rate increases and magnesium combines with ammonia to magnesium hydrogen carbonate ($Mg(HCO_3)_2$), even if phosphoric acid does not take part in the reaction. When the pH is equal to 9, the concentration of nutrient salts in lake water is decreasing rapidly and is converted into a crystallized form as a magnesium ammonium phosphate (MAP, $MgNH_4PO_4 \cdot 6H_2O$) as in Eq. (2)[4]. In the case of Eq. (3), it is well known that the reaction only proceeds in a condition of pH 11; namely, high concentrations of magnesium ion and hydroxide ion are present in the lake water, and they are combined to form magnesium hydroxide ($Mg(OH)_2$)[5].

### Setup Condition on Microcosm for the Elution of Nutrient Salts

The sediment and the lake water are sampled from Lake Tega located in northwest area of Chiba prefecture, Japan.

Sediment improvement effect for the DAF and CRM methods is evaluated in physical, chemical, and biological measurement values with glass vessels (14-cm height, 7.2-cm diameter and 470-ml volume) as a microcosm filled with sediment and lake water. The volume of microcosm in this study is considered to be a reasonable size for the chemical and biological estimation of sediment improvement effect. After the sediment is purified with DAF method, the fine particles of MgO are sprinkled on the surface of the sediment layers (Run 3, Run 4, and Run 5 in Table 1), which are set up in glass vessels for microcosm. The vessels for culture are kept on 20˚C for 28 days and set up in two groups: dark and light conditions.

**TABLE 1**
**Cultural Condition in Experiment**

| Run No. | Method | Materials ($g/m^2$) |
|---|---|---|
| Run1 | Raw mud | — |
| Run2 | DAF | — |
| Run3 | DAF | MgO 100 |
| Run4 | DAF | MgO 400 |
| Run5 | DAF | MgO 2,000 |

## RESULTS AND DISCUSSION

### Sediment Remediation in Microcosm

The restraint effects on total nitrogen (T-N) and total phosphorus (T-P) related to the elution of nutrient salts under the dark conditions are shown in Fig. 2 and Fig. 3. Here, the symbols in both figures show the average value of each experimental condition: raw/nontreated sediment, remedied sediment with DAF, and elution control with CRM, respectively. The suffix "o" means the concentration value on raw/lake water at starting elapsed time, "r" and "t" mean the concentration values on the conditions of nonremediation and remedied sediment.

In all cultural systems of the total nitrogen in Fig. 2, the tendency is for total nitrogen concentration to decrease for about 10 days of elapsed time, and after 10 days the tendency is to increase on Run 4 and Run 5. Because the amounts of MgO in Run 4 and Run 5 (400 $g/m^2$ and 2,000 $g/m^2$, respectively) produce a reduction state in the sediment layer, the elution of total nitrogen is greater than Run 2 and Run 3. On the other hand, for elution of phosphorus in Fig. 3, the tendency for decreasing the total phosphorous is confirmed in each experimental condition.

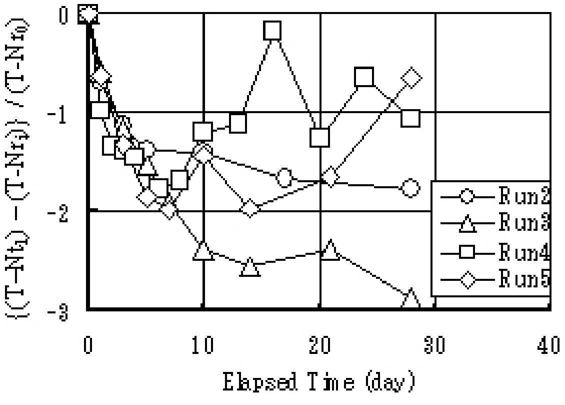

FIGURE 2. Restraint effect on total nitrogen (T-N).

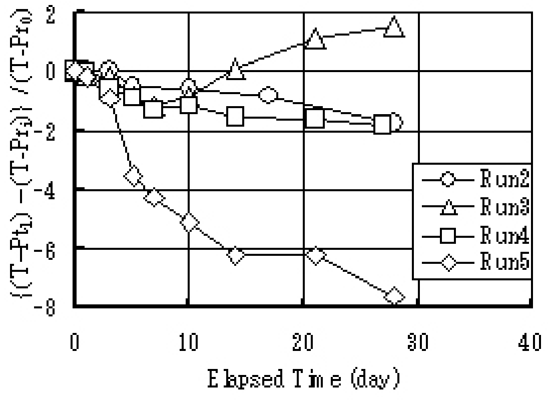

FIGURE 3. Restraint effect on total phosphorus (T-P).

As a result, it is reasonable to consider that the magnesium ammonium phosphate (MAP) shown in Eq. 2 is formed on the surface of the sediment layer until 10 days of elapsed time, because of MgO sprinkled on the surface with CRM. After the tenth day, especially in Run 4 and Run 5, the magnesium phosphate is formed as the result of the reaction in Eq. 3.

### Effect of Sediment Treatment Considering Phytoplankton

The relationship between T-N and T-P in light condition is shown in Fig. 4. The solid line and dotted lines in the figure represent the empirical component ratios of a biology cell: The optimum ratios for N/P regarding water bloom, the ten times line, and the Redfield index for the marine algae are 13.5, 10.0, and 7.2, respectively. Data for Lake Tega are plotted in the large area, as compared with the data of Lake Kojima, Lake Kasumi, Lake Suwa, and Lake Biwa, which are distributed near the point of intersection of the coordinate axes. Furthermore, it can be clearly recognized that the data remedied by DAF and CRM are plotted near the coordinate axes, and that the purification proceeds from nonremedied condition to DAF and from DAF to the hybrid remediation with DAF and CRM.

Purified concentration obtained by photosynthetic microbiology with DAF and CRM remediation is measured as T-N and T-P in Fig. 5. Here, the data are calculated as the difference of concentrations under dark and light control conditions in the glass vessels, and the suffixes “D”, “L”, and “0” regarding T-N and T-P on the each coordinate axis mean dark condition, light condition, and initial concentrations, respectively, of the vessel water, i.e., the lake water. It can be clearly observed that almost of data are distributed in an area of the third quadrant, except of a part of the data on Run 2, and that in the case of the nonremedied experiment run, Run 1, the data show a relatively high removal ratio. However, the high removal of Run 1 could occur due to an abnormal multiplication and an extinction/settling of phytoplankton, because pH value is continuously kept at the high level, from 8 to 9.5, during the experimental period. This phenomenon represents the expanded ecological circulation; therefore, it is not connected to the subject of lake water purification. The shadow partition shown in Fig. 5 is an indication of the same amount of removal as an initial concentration of vessel water.

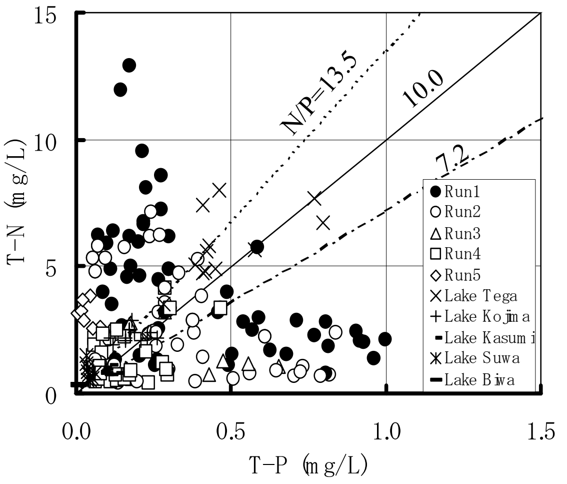

**FIGURE 4.** Relationship between T-N and T-P on the light condition.

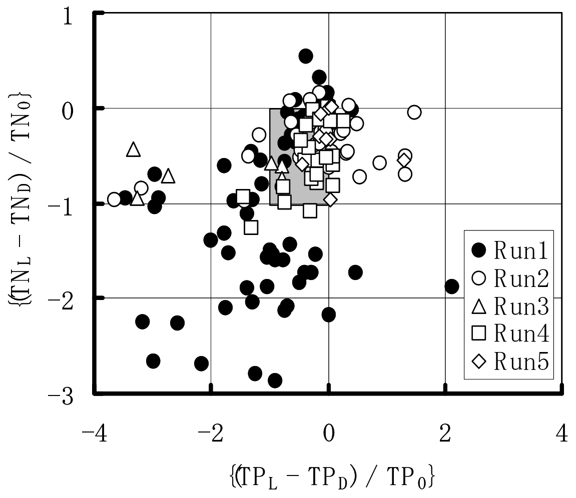

**FIGURE 5.** Purified concentration due to photosynthetic microbiology.

### Quantitative Evaluation on the Remediation Methods

The comparison of the methods, DAF, CRM, and their hybrid is conducted with plastic vessels (height: 100 cm, diameter: 30 cm) as a microcosm filled with sediment (thickness: 10 cm) and lake water (water volume: 56 ls). They are set onsite at the lake and are naturally controlled in sunlight, temperature, etc. An aphotic zone (30 cm) in the vessels is made in the lower side of the vessel by the insulation and blind sheet.

The result for remediation effect is shown in Fig. 6(a) and (b) as water quality, and in (c) and (d) as phytoplankton species, in summer and winter seasons, respectively. Here, C and N in the vertical axes are the concentration of water quality and the cell number of plankton species. The suffixes "i" and "j" are the terms of water quality and the cultural conditions in the experiment, respectively. Furthermore, the suffix "0" means the concentration of each term in raw water. The value of remediation effect ratio in the vertical axis can be defined as 1.0 in a case of noneffective condition.

In all remediation methods of the dissolved total nitrogen (DT-N) and $PO_4$-P in Fig. 6, it is clearly determined that the high efficiency of remediation is observed in the figure as about 0.5 times that of the nonremedied water quality. Namely, it is determined that the concentration of the water quality in the lake is reduced to half the value of the nontreated sediment condition. On the other hand, a high concentration of chlorophyll-a is noticed in the hybrid treatments of Run 5 in summer and of Run 4 in winter, by reason of the height transparency and elution of nitrogen from the sediment, due to DAF treatment and MgO sprinkling treatment.

The remediation effect on the aquatic plankton, such as blue algae, green algae, and diatoms in each treatment methods is drawn in Fig. 6(c) and (d). First, it is recognized that the number of colonies of blue algae is gradually decreased to one-fourth the number of the nonremedied water with DAF and MgO treatments in the summer case. In the case of MgO-2000 as Run 5, a large number of cells of green algae and diatoms are represented in summer by extremely high N/P ratio. A ratio of more than 1.5 in the green algae is counted in Run 2 and 4 of the winter case. And the number of diatom cells measured is almost steady in spite of DAF treatment (Run 2), MgO-400 treatment (Run 4), and the nontreatment (Run 1).

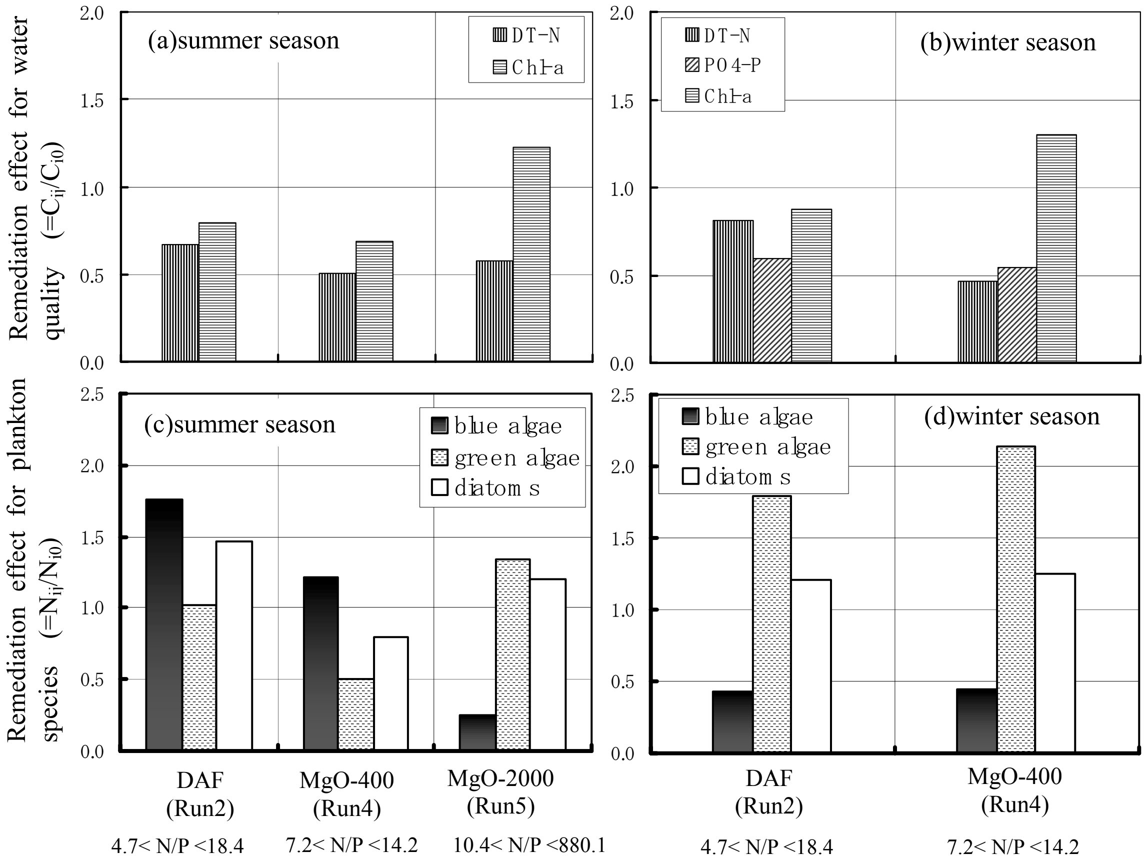

**FIGURE 6.** Remediation effect for water quality and phytoplankton species.

From the viewpoint of biological water quality index[6], the decreasing of dominant phytoplankton such as blue algae, which forms "water bloom" and produces toxins such as microcystin and anatoxin-*a*, means the increasing of remediation effect in the lake, and the shifting from eutrophic state (Run 1) to mesotrophic and/or oligotrophic state (from Run 2 to Run 5).

### CONCLUSION

The effects of sediment remediation methods are studied in this paper, by means of reducing the size of ecological circulation and for the elution control, using a hybrid method between Dissolved Air Flotation (DAF) and Chemical Remediation Materials (CRM). The results are as follows:

1. Hybrid system with DAF and CRM for the remediation of eutrophic sediment has two efficiencies for the purification, one is the washout effect for the nutrient salts with DAF method, and the other is elution control with CRM method.
2. The mechanism for the elution control is to produce the reactions of magnesium ammonium phosphate (MAP) and magnesium phosphate by sprinkling the MgO particles on the sediment layer.
3. It is possible to state that the hybrid system is a reasonable method to reduce the size of ecological circulation, and to lead to a normal/quasi-oligotrophic condition for an aqua-ecological system.

4. The sediment remediation treatment, such as DAF and CRM, can lead to the shifting of dominant phytoplanktonic flora from blue algae to green algae and/or diatoms and the decreasing of concentration for the eutrophic matters into about 0.5 times of raw water.

### ACKNOWLEDGMENT

This work is partially supported by the Foundation of River and Watershed Environment Management of Japan.
